# Streamlined Surgical Draping Reduces Subcutaneous Implantable Cardioverter-defibrillator Implant Procedure Preparation Time

**DOI:** 10.19102/icrm.2018.090705

**Published:** 2018-07-15

**Authors:** Marcy Mcqueen, Lori A. Woodford, Eric Holick, Kevin Wolfer, Anish K. Amin

**Affiliations:** ^1^Section of Cardiac Electrophysiology, Department of Cardiology, OhioHealth Heart & Vascular Physicians, Riverside Methodist Hospital, Columbus, OH, USA

**Keywords:** *S-ICD*, subcutaneous ICD, subq ICD, surgical draping

## Abstract

The subcutaneous implantable cardioverter-defibrillator (S-ICD) is a proven alternative to transvenous implantable cardioverter-defibrillator systems. One critique of S-ICD use, however, has been the time required for implantation. Here, we discuss the use of an alternative surgical draping technique to reduce preparation time for device implantation.

## Introduction

The subcutaneous implantable cardioverter-defibrillator (S-ICD) is a proven alternative to transvenous implantable cardioverter-defibrillator (ICD) systems.^1,2^ One critique of S-ICD use, however, has been the time required for implantation. Though the two-incision technique has reduced the time needed for implantation, overall laboratory utilization remains significant as compared with during transvenous ICD implant.^3^ Implantation of an S-ICD requires extensive surgical preparation to accommodate the multiple incisions. Traditional methods of surgical site preparation cover the entire thorax from the sternal notch to the naval. These techniques require two scrub nurses to adequately cleanse, prepare, and drape the entire region. Relative to transvenous ICD implant preparation, this demands more intensive resources in terms of both time and nursing support.

## Methods

We retrospectively analyzed the implants of 58 consecutive S-ICD patients between 2014 and 2017. The patient cohort was 70.6% male and 29.4% female. Body mass index (BMI) ranged from 22.1 kg/m^2^ to 68.4 kg/m^2^.

The procedure logs were reviewed for the time at which body surface preparation was completed using either ChloraPrep™ (Becton Dickinson, Franklin Lakes, NJ, USA)^4^ or Betadyne^®^ (Purdue Pharma LP, Stamford, CT, USA) and then examined for the time at which draping of the patient was complete. The total drape time was calculated as the difference between the time at which draping was complete and the time at which body surface preparation was complete, minus a three-minute allowance for drying of the preparation agent.

**Figure 1:**
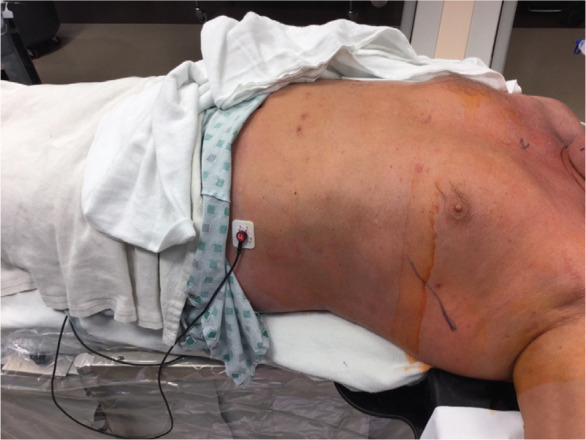
Example of a patient prepared with chlorahexidine scrub from the sternal notch to the umbilicus and from the right sternal border to the left posterior axillary line and left axilla to the elbow.

**Figure 2:**
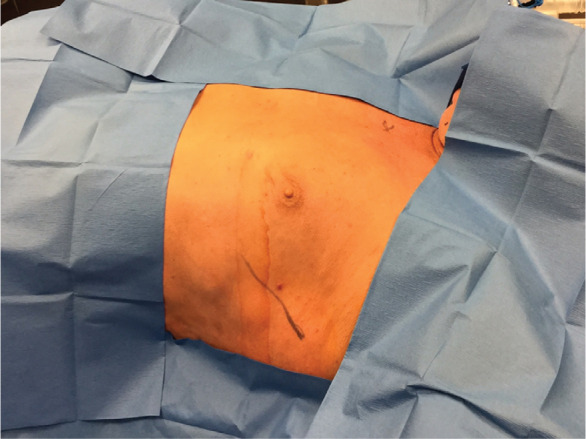
Four small surgical drapes were placed on the surgical prep site border. The area was then subsequently covered with a 3M™ Ioban™ 2 Antimicrobial Incise Drape (3M, St. Paul, MN, USA).

**Figure 3:**
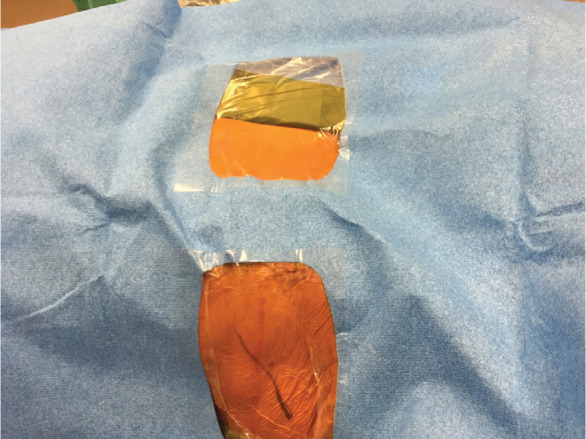
A standard pacemaker drape was placed with the first window at the device pocket and opened towards the sternum and with the second window allowing access to the xyphoid for the second incision.

Traditional operating room (OR) preparation consisted of a full cardiac surgery surface preparation followed by draping involving two scrubbed personnel to cover a field from the neck through the navel. The alternate technique consisted of body surface preparation of the midaxillary line and the subxiphoid space with a single scrub person and placement of a readily available pacemaker drape (Convertors^®^ Pacemaker Sheet D231OC; Cardinal Health, Dublin, OH, USA). The new draping process was completed in three easy steps after marking S-ICD system placement, as follows: (1) the body surface was prepared from the sternal notch to the umbilicus and from the right sternal border to the left posterior axillary line and left axilla to the elbow **(Figure 1)**; (2) four small surgical drapes were placed on the surgical prep site border and covered with a 3M™ Ioban™ 2 Antimicrobial Incise Drape (3M, St. Paul, MN, USA) **(Figure 2)**; and (3) a standard pacemaker drape was placed with the first window at the device pocket and opened towards the sternum and with the second window allowing access to the xyphoid for the second incision **(Figure 3)**.

A univariate analysis was completed to demonstrate independence of procedural drape time as a continuous variable. The procedure drape time was compared using Welch’s two-sided t-test between the standard OR preparation and drape scenario and the alternative preparation and drape scenario.

## Results

**Figure 4:**
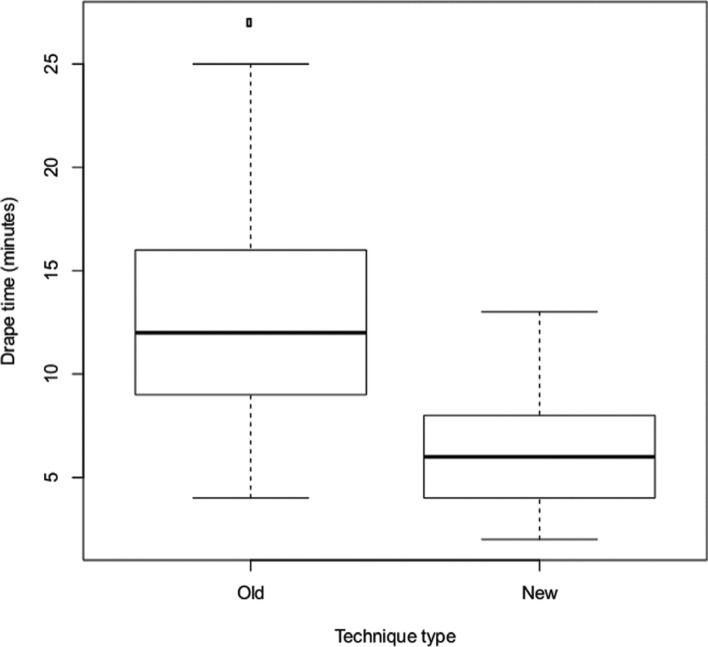
Drape time by technique type.

Twenty-four of the 58 patients were covered using the standard OR drape technique. The remaining 34 patients were managed using the alternate technique. Drape time was an independent variable across all demographics **(Figure 4)**. The average time required to drape patients using the traditional OR protocol was 13.4 minutes, with a standard deviation of 6.6 minutes. This is in comparison with the average time required to drape patients using the alternative approach, which was 5.8 minutes with a standard deviation of 2.9 minutes, representing a statistically significant difference (p < 0.001). This represents a total time savings of 7.6 minutes using the alternate draping technique **(Figure 4)**. All patients received the EMBLEM™ MRI S-ICD system (Boston Scientific, Natick, MA, USA).

**Figure 5:**
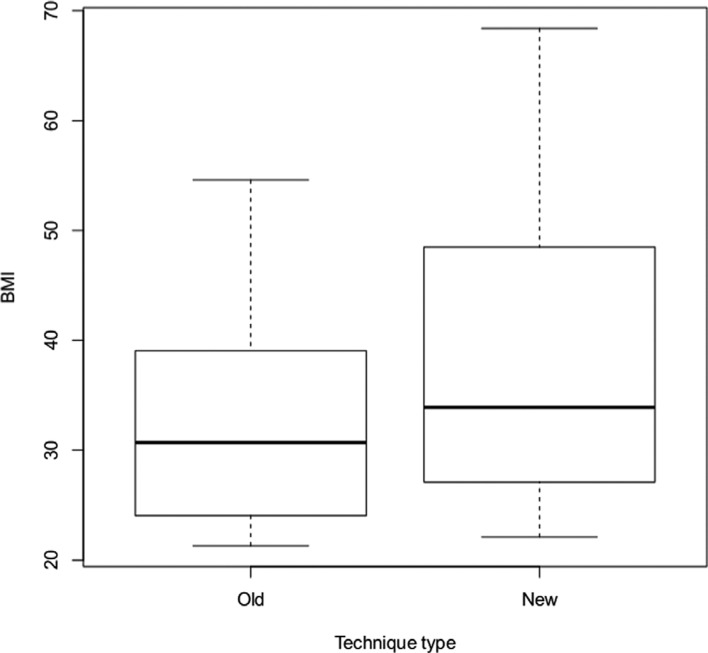
BMI by technique type.

BMI had no significant effect on the time for draping or the ability to place an alternate drape. The average BMI in the standard drape group was 32 kg/m^2^ versus 38 kg/m^2^ in the alternate drape group **(Figure 5)**. Procedure time similarly demonstrated no significant difference between the two groups relative to BMI. Average procedure time for the traditional OR drape group was 162 minutes versus 143 minutes for the alternate group.

Eighteen patients had inadequate procedure logs and were removed from our final assessment. The only reason for exclusion was an inability to discern a clear time of drape in the procedure log. Six of these patients underwent a standard OR drape technique, while the remaining 12 underwent the alternative drape technique.

## Discussion

The S-ICD is a reasonable alternative to traditional transvenous ICD implantation and, in many cases, is a more favorable option for patients.^5^ Time of implantation has been described as being longer than transvenous ICD implantation and remains a hurdle for widespread adoption. Improved implantation techniques and operator experience have demonstrated considerable reduction in total operator-dependent procedure times.^1–3^ Within the electrophysiology laboratory or OR, the traditional cardiac surgery drape technique remains a considerable burden on laboratory resources, including with respect to the number of surgical scrub personnel required for draping as well as time spent in the laboratory. Our assessment demonstrates that novel drapes and techniques can be utilized to relieve both surgical personnel and laboratory in-room time.

BMI should not limit the use of the described alternative drape technique, as demonstrated by the achievement of a successful drape and implant procedure with patients with a broader BMI range in our analysis **(Figure 5)**. The drape allows for flexibility in body size by condensing or expanding the fold between the two surgical sites as necessary.

### Study limitations

Our study is limited by its retrospective nature, although the preparation and drape times were collected prospectively. The small number of patient procedure logs able to be assessed secondary to inadequate timestamping of portions of the procedure also hindered the overall analysis, as did relying on the presumed accuracy of the procedure log. Nonetheless, with a small dataset, we were still able to demonstrate the significance of this novel clinical preparation method.

## Conclusions

The introduction of improved techniques and tools for S-ICD implantation has markedly reduced procedure times but done little to reduce the in-laboratory preparation and care duration of the patient. Procedure efficiency must also recognize the burden of care required in the laboratory or OR, as well as the total number of laboratory personnel required. Utilizing a readily available pacemaker drape, we have demonstrated a significant reduction in patient preparation time and reduced the overall number of laboratory personnel required to facilitate the implantation of an S-ICD.

## References

[1] Weiss R, Knight BP, Gold MR (2013). Safety and efficacy of a totally subcutaneous implantable-cardioverter defibrillator. Circulation..

[2] Boersma L, Barr C, Knops R (2017). Implant and midterm outcomes of the subcutaneous implantable cardioverter-defibrillator registry: the EFFORTLESS study. J Am Coll Cardiol..

[3] Brouwer TF, Miller MA, Quast ABE (2017). Implantation of the subcutaneous implantable cardioverter-defibrillator: an evaluation of 4 implantation techniques. Circ Arrhythm Electrophysiol..

[4] Becton Dickinson ChloraPrep™ in-service resources.

[5] Basu-Ray I, Lui J, Jia X (2017). Subcutaneous versus transvenous implantable cardioverter defibrillator therapy: a meta-analysis of case-control studies. JACC Clin Electrophysiol..

